# Analysis of current status of knowledge, attitudes, and influencing factors regarding chikungunya fever among medical staff in Chengdu, Sichuan province, China: a cross-sectional study

**DOI:** 10.3389/fpubh.2026.1751203

**Published:** 2026-07-01

**Authors:** Qin Zhang, Yiting Du, Mei Cha, Hairong Wang, Erdan Luo, Zhenke Zhou, Juan Xiang, Yao Luo, Xiaodan Gu, Lei Li, Zheng Li, Deyong Zhou, Rui Shen, Hui Wang, Cheng Xie, Meiyan Ni, Yu Zhou, Jingrong Yang, Jinghua Ye, Xiaoli Jiang, Yonghong Lin

**Affiliations:** 1 Department of Public Health, Chengdu Women’s and Children’s Central Hospital, School of Medicine, University of Electronic Science and Technology of China, Chengdu, Sichuan, China; 2 Emergency Department, Chengdu Women’s and Children’s Central Hospital, School of Medicine, University of Electronic Science and Technology of China, Chengdu, Sichuan, China; 3 Jianyang Center for Disease Control and Prevention, Chengdu, Sichuan, China; 4 GCP Institution, Chengdu Women’s and Children’s Central Hospital, School of Medicine, University of Electronic Science and Technology of China, Chengdu, Sichuan, China; 5 Emergency Department, Chengdu Seventh People’s Hospital, Chengdu, Sichuan, China; 6 Emergency Department, Chengdu Sixth People’s Hospital, Chengdu, Sichuan, China; 7 Emergency Department, The Public Health Clinical Center of Chengdu, Chengdu, Sichuan, China; 8 Emergency Department, No. 1 Orthopedic Hospital of Chengdu, Chengdu, Sichuan, China; 9 Department of Pediatrics, Chengdu Second People’s Hospital, Chengdu, Sichuan, China; 10 Emergency Department, Chengdu Longquanyi District Maternal and Child Health Hospital, Chengdu, Sichuan, China; 11 Department of Pediatrics, Hanyuan County People’s Hospital, Ya’an, Sichuan, China; 12 Department of Pediatrics, Shuangliu District Traditional Chinese Medicine Hospital, Chengdu, Sichuan, China; 13 Department of Pediatrics, The Third People’s Hospital of Chengdu, Chengdu, Sichuan, China; 14 Emergency Department, Sichuan Provincial Maternity and Child Health Care Hospital, Chengdu, Sichuan, China; 15 Department of Pediatrics, Chengdu Integrated TCM & Western Medicine Hospital (Chengdu First People’s Hospital), Chengdu, Sichuan, China; 16 Emergency Department, Chongzhou People’s Hospital, Chongzhou, Sichuan, China; 17 Department of Pediatrics, Xindu District People’s Hospital, Chengdu, Sichuan, China; 18 Laboratory Department, Pengzhou Maternal and Child Health Care Hospital, Chengdu, Sichuan, China; 19 Department of Gynecology, Chengdu Women’s and Children’s Central Hospital, School of Medicine, University of Electronic Science and Technology of China, Chengdu, Sichuan, China

**Keywords:** attitude, chikungunya fever, knowledge, medical personnel, training

## Abstract

**Background:**

Chikungunya (CHIK) is an emerging mosquito-borne disease with increasing importation risk in China. In non-endemic settings, delayed detection may facilitate local transmission, making healthcare workers’ knowledge and attitudes critical for rapid response.

**Objective:**

To assess CHIK-related knowledge and attitudes among medical personnel in Chengdu, Sichuan Province, and identify factors associated with positive prevention attitudes.

**Methods:**

A cross-sectional, self-administered online survey was executed from July 30 to August 6, 2025, targeting medical workers across 20 healthcare institutions in Chengdu using the Wenjuanxing platform. The questionnaire gathered demographic information, CHIK-related training exposure (yes/no), and assessments of knowledge and attitudes. Knowledge was assessed using 15 items (range 0–15; 1 point awarded for each right response). Attitudes were evaluated using Likert-type items; a cumulative attitude score of ≥20 signified a favorable attitude. Group disparities were analyzed with Chi-square tests alongside suitable parametric and nonparametric assessments. Hierarchical logistic regression (Block 1: demographics/occupational characteristics; Block 2: training; Block 3: knowledge score) was employed to ascertain factors correlated with positive attitudes.

**Results:**

1,092 questionnaires were included, yielding an effective response rate of 90.10%; 83.70% indicated they had received training relevant. The average knowledge score was 11.66 ± 2.15. Despite high performance in primary prevention and clinical management, significant deficiencies were noted in early detection and surveillance-related operational knowledge: merely 42.58% accurately interpreted the RT-PCR Ct criterion, 54.85% recognized suspected cases, 56.14% comprehended diagnostic criteria, and 59.80% were aware of the 24 h online reporting requirement. Knowledge scores were elevated among individuals with advanced education, physicians, tertiary hospital personnel, and those who had training (*p* < 0.05). Attitude scores varied according to age, job type, hospital type, practice category, years of experience, and professional title (*p* < 0.05). In hierarchical logistic regression, training (OR = 3.971, *p* < 0.001) and elevated knowledge scores (OR per point = 1.130, *p* < 0.001) were independently linked to a favorable prevention-and-control attitude.

**Conclusion:**

Chengdu medical personnel demonstrated general CHIK knowledge and positive attitudes, yet significant operational gaps persist in early detection and reporting protocols. Training and knowledge are key determinants of favorable attitudes, highlighting the need for standardized, process-oriented training emphasizing diagnostic and reporting procedures.

## 1 Introduction

Chikungunya fever (CHIK) is an acute mosquito-borne infectious disease induced by the chikungunya virus (CHIKV), spread by the bites of *Aedes aegypti and Aedes albopictus* (1–5). CHIKV has disseminated to more than 110 countries and areas worldwide. The clinical manifestations of chikungunya include abrupt onset of high fever, severe arthralgia, and rash; in some patients, persistent arthritis may substantially impair quality of life and social functioning (6–8). Although chikungunya is often regarded as a self-limiting disease, severe complications and excess mortality have been reported during major outbreaks, indicating that its public health impact may be underestimated in some settings (7, 8). Recently, the rise in international travel and trade has increased the likelihood of imported CHIK cases in non-endemic locations, contributing to sporadic local transmission or outbreaks in multiple regions (8, 9). Since the initial report of an imported CHIK case in China in 2008, numerous imported and locally transmitted cases have been subsequently detected in Guangdong, Zhejiang, Yunnan, and other locations (10, 11). In July 2025, a significant outbreak of imported chikungunya fever transpired in Foshan City, Guangdong Province, China, highlighting the potential for rapid amplification once ecological and operational conditions align (12).

Chengdu, being a transportation nexus and demographic focal point in southwest China, annually draws significant human migration from Southeast Asian and South Asian nations. The local warm and humid climate creates an optimal setting for the proliferation of *A. albopictus*, presenting a possible danger of local transmission initiated by imported cases (13). Moreover, data indicates that the climatic adaptability and geographic distribution of *Aedes vectors* in China are expanding due to changing environmental conditions, thereby heightening the risk of local transmission following importation (14). Medical personnel, as primary agents in epidemic prevention, control, and clinical treatment, possess knowledge and attitudes regarding the epidemiological characteristics, critical diagnostic and therapeutic strategies, and preventive measures for CHIK that directly influence the efficiency and efficacy of emergency epidemic responses. Prior research in various contexts has indicated deficiencies in healthcare personnel’ understanding of arboviruses and their risk assessment, proposing that specialized training correlates with enhanced readiness (15). To our knowledge, there have been no documented systematic surveys regarding the knowledge and attitudes of medical personnel in Chengdu. This study seeks to (i) evaluate the existing knowledge and attitudes toward prevention and control of CHIK among medical workers in Chengdu, Sichuan Province, China, and (ii) identify demographic and occupational factors linked to more favorable prevention and control attitudes. This study is essential for evaluating the local medical prevention and control system’s readiness for CHIK, pinpointing knowledge and capability gaps concerning CHIK, and formulating focused training initiatives and emergency response measures.

## 2 Materials and methods

### 2.1 Study design and setting

This was a cross-sectional survey conducted in Chengdu, Sichuan Province, China. Medical personnel from 20 healthcare facilities in Chengdu were invited to participate. The online survey was administered from July 30 to August 6, 2025, using the Wenjuanxing platform.

### 2.2 Participants and sample size

Healthcare professionals of diverse ranks employed in the participating medical facilities were invited. A total of 1,212 questionnaires were gathered throughout the survey period; following data quality assessments (e.g., incomplete or invalid responses), 1,092 questionnaires were incorporated into the final analysis, resulting in an effective response rate of 90.10%.

### 2.3 Survey instrument

A self-constructed questionnaire was developed based on a review of relevant literature, covering basic information and CHIK-related knowledge, attitudes, and preparedness for prevention and control. The questionnaire underwent expert content validation by four experts (three clinical medical professionals and one hospital administrator) across five dimensions (relevance, comprehensiveness, representativeness, appropriateness of alternatives, and ethical compliance). All items had an I-CVI > 0.80, and the overall S-CVI was 0.95. A pilot survey was conducted among 100 medical personnel prior to the formal survey. The construct validity and reliability were satisfactory (KMO = 0.851; Bartlett’s test *χ*^2^ = 230.463, *p* < 0.001; total Cronbach’s *α* = 0.870).

### 2.4 Measures and scoring

A cumulative knowledge score was determined by assigning 1 point for each correct answer, with a theoretical range of 0 to 15 points.

Attitudes toward CHIK prevention and control were evaluated via Likert-type measures. Responses of ≥4 on the Likert scale for each question were categorized as “agree/strongly agree”; hence, a cumulative attitude score of ≥20 was designated as indicative of a positive prevention-and-control attitude. The binary indicator (positive versus non-positive attitude) served as the dependent variable in logistic regression studies. Training was assessed by a self-reported binary question enquiring if the respondent had ever undergone any CHIK-related training. The questionnaire did not gather specific details regarding training type (e.g., online/offline), location, or precise timing; hence, training was assessed as a binary exposure variable.

### 2.5 Data collection procedure

The survey was conducted online through the Wenjuanxing platform. Before completing the questionnaire, all participants were informed of the study objectives, the anonymous nature of the survey, and the voluntary nature of participation. Submission of the completed questionnaire was regarded as provision of informed consent. All responses were collected during the study period, and questionnaires were screened for completeness and validity before analysis.

### 2.6 Statistical analysis

The database was established, and data analysis was conducted using IBM SPSS Statistics 29 software. The validity and reliability of the questionnaire were assessed using Cronbach’s *α* coefficient, the KMO test, and Bartlett’s spherical test. Quantitative data were presented as mean ± standard deviation (*M* ± SD) or median with interquartile range (IQR), based on their distribution. The normality of data distribution was assessed using the Shapiro–Wilk test. Given the non-normal distribution of knowledge and attitude scores, non-parametric tests (Mann–Whitney *U* and Kruskal–Wallis *H*) were primarily applied for group comparisons of these scores. Categorical data were expressed as frequency and percentage (*n*, %), and the Chi-square test was employed to examine disparities in the training rate and awareness rate of CHIK among medical personnel with varying demographic features. Pairwise comparisons for multi-group categorical variables exhibiting significant differences were conducted utilizing Bonferroni correction. For group comparisons of normally distributed data, the *t*-test or analysis of variance (ANOVA) was employed to evaluate the knowledge levels and attitude scores on CHIK among medical personnel with varying demographic features, and pairwise comparisons were performed using the SNK test or Dunnett-*t* test, contingent upon the homogeneity of variance. Non-parametric tests were used for group comparisons of non-normally distributed data, the Mann–Whitney *U* test was employed for comparisons between two groups, and the Kruskal-Wallis H test for comparisons across more than two groups. If the Kruskal-Wallis *H* test indicated a statistically significant difference, post-hoc pairwise comparisons were conducted using the Mann–Whitney *U* test with a Bonferroni correction applied to adjust the significance level for multiple testing. The chi-square test was employed to examine the relationship between medical personnel’s awareness of CHIK and their attitudes toward prevention and control. Correlation between continuous variables were assessed using bivariate linear correlation. Hierarchical (block-wise) logistic regression was employed to investigate factors correlated with a positive prevention-and-control attitude (positive versus non-positive). A *p*-value less than 0.05 was deemed statistically significant.

## 3 Results

### 3.1 Demographic attributes of the survey population

The majority of respondents were female nurses employed at tertiary hospitals, with most possessing a bachelor’s degree (Table 1). We acquired a total of 1,092 valid questionnaires throughout this survey, culminating in an effective response rate of 90.10%. The mean age of the questioned medical personnel was 35.23 years, with median age 34.00 (29.00, 39.00) years old, predominantly female (84.43%) and possessing a bachelor’s degree (72.16%). Nurses (52.84%) and doctors (38.00%) constituted the predominant share of job positions. The majority of respondents were employed in tertiary hospitals (85.35%), predominantly in general hospitals (51.92%) and maternal and child specialist hospitals (40.75%). The primary departmental distribution comprised high-risk clinical departments (38.55%), comprising emergency departments and fever clinics, and specialized population departments (29.21%), encompassing pediatrics and obstetrics and gynecology. The predominant practice category was Western medicine, comprising 75.37%. The mean duration of professional experience was 11.74 years, with more than half (53.57%) possessing fewer than 10 years of experience. Intermediate (45.42%) and primary (36.45%) titles were the most prevalent in terms of professional designations.

**Table 1:** Demographic characteristics of respondents (*N* = 1,092).

Demographic characteristics	Number (%)
Gender	
Female	922 (84.43)
Male	170 (15.57)
Age	
≤30 years	341 (31.23)
31–40 years	513 (46.98)
≥41 years	238 (21.79)
Educational background	
Junior college or below	157 (14.38)
Bachelor’s degree	788 (72.16)
Master’s degree or above	147 (13.46)
Job type	
Doctor	415 (38.00)
Nurse	577 (52.84)
Others	100 (9.16)
Hospital level	
Tertiary hospital	932 (85.35)
Secondary hospital	160 (14.65)
Hospital type	
General hospital	567 (51.92)
Maternal and child specialist hospital	445 (40.75)
Other specialist hospitals	80 (7.33)
Department	
High-risk clinical departments (emergency + fever clinic)	421 (38.55)
Special population departments (pediatrics + obstetrics and gynecology)	319 (29.21)
General clinical departments^*^	159 (14.56)
Non-clinical departments (medical technology + administration and logistics)	193 (17.68)
Clinical practice category	
Western medicine	823 (75.37)
Public health	210 (19.23)
Others	59 (5.40)
Years of working experience	
≤10 years	585 (53.57)
11–20 years	358 (32.78)
≥21 years	149 (13.65)
Professional title	
Uncertified	42 (3.85)
Primary	398 (36.45)
Intermediate	496 (45.42)
Senior	156 (14.28)

*General clinical departments are medical units with low risk of exposure to infectious diseases that serve the general adult population, including internal medicine, surgery, specialties, and outpatient services.

### 3.2 Involvement in CHIK training

Of all respondents, 914 (83.70%) had received CHIK-related training. Training coverage was generally high; however, uptake exhibited a slight variation based on institutional and professional factors—being lower among personnel in secondary hospitals and among those lacking a professional title, as well as lower in respondents holding a master’s degree or higher compared to those with a bachelor’s degree (Table 2).

**Table 2:** Participation in CHIK training by respondent characteristics (*N* = 1,092).

Demographic characteristics	Total number	Number of trained participants (%)	95% CI	*χ* ^2^	*p*
Gender				3.509	0.061
Female	922	780 (84.60)	(82.26, 86.93)		
Male	170	134 (78.82)	(72.62, 85.03)		
Age				2.609	0.271
≤30 years	341	278 (81.52)	(77.38, 85.66)		
31–40 years	513	430 (83.82)	(80.62, 87.02)		
≥41 years	238	206 (86.55)	(82.19, 90.92)		
Educational background				10.51	**0.005**
Junior college or below	157	130 (82.80)	(76.83, 88.77)		
Bachelor’s degree	788	674 (85.53)	(83.07, 87.99)		
Master’s degree or above	147	110 (74.83)	(67.73, 81.93)		
Job type				2.193	0.334
Doctor	415	353 (85.06)	(81.62, 88.50)		
Nurse	577	482 (83.54)	(80.50, 86.57)		
Others	100	79 (79.00)	(70.88, 87.12)		
Hospital level				4.27	**0.039**
Tertiary hospital	932	789 (84.66)	(82.34, 86.97)		
Secondary hospital	160	125 (78.13)	(71.65, 84. 60)		
Hospital type				2.734	0.255
General hospital	567	469 (82.72)	(79.59, 85.84)		
Maternal and child specialist hospital	445	373 (83.82)	(80.39, 87.26)		
Other specialist hospitals	80	72 (90.00)	(83.28, 96.72)		
Department				1.721	0.632
High-risk clinical departments	421	359 (85.27)	(81.87, 88.67)		
Special population departments	319	261 (81.82)	(77.56, 86.07)		
General clinical departments	159	134 (84.28)	(78.56, 90.00)		
Non-clinical departments	193	160 (82.90)	(77.54, 88.26)		
Practice category				5.4	0.067
Western medicine	823	695 (84.45)	(81.97, 86.93)		
Public health	210	176 (83.81)	(78.79, 88.83)		
Others	59	43 (72.88)	(61.20, 84.57)		
Years of working experience				4.456	0.108
≤10 years	585	477 (81.54)	(78.39, 84.69)		
11–20 years	358	310 (86.59)	(83.05, 90.14)		
≥21 years	149	127 (85.23)	(79.47, 91.00)		
Professional title				9.864	**0.020**
Uncertified	42	28 (66.67)	(51.80, 81.53)		
Primary	398	339 (85.18)	(81.67, 88.68)		
Intermediate	496	414 (83.47)	(80.19, 86.75)		
Senior	156	133 (85.26)	(79.63, 90.88)		

Bold values indicate statistically significant results (*p* < 0.05).

### 3.3 Medical personnel’s knowledge of CHIK

The scoring range for medical personnel was 2–15 points, with a mean score of 11.66 ± 2.15 points, a median score of 12.00 (10.00, 13.00). Nevertheless, cognitive performance was least effective for operational items inside the monitoring support system and early detection. Specifically, accurate replies were minimal for RT-PCR Ct interpretation (Q12) and only moderately satisfactory for suspected/confirmed case definitions (Q3, Q5) and reporting timelines (Q14) (Tables 3, 4). Knowledge ratings varied among subgroups, exhibiting a structural gradient based on education, employment function, hospital level, and department (Table 5).

**Table 3:** Medical personnel’s cognition of chikungunya fever (CHIK): correct response rates for key knowledge items (*N* = 1,092).

Question no.	Question content	Number of correct answers (correct rate %)
Q1	Main transmission routes of CHIK	1,078 (98.72)
Q2	Typical clinical manifestations of CHIK	906 (82.97)
Q3	Conditions for diagnosing suspected CHIK cases	599 (54.85)
Q4	Key differential points between CHIK and dengue fever	841 (77.01)
Q5	Diagnostic basis for confirmed CHIK cases (Multiple Choice)	613 (56.14)
Q6	Time limit for mosquito-proof isolation of CHIK patients	927 (84.89)
Q7	Treatment principles for CHIK	850 (77.84)
Q8	Main sources of CHIK infection	968 (88.64)
Q9	Class I areas in China with high CHIK epidemic risk	1,037 (94.96)
Q10	Most critical prevention and control measures when emergency departments receive suspected CHIK patients	1,039 (95.15)
Q11	Incubation period of CHIK	756 (69.23)
Q12	Positive criteria for detecting Chikungunya virus nucleic acid using real-time RT-PCR	465 (42.58)
Q13	High-risk groups for poor prognosis of CHIK	1,003 (91.85)
Q14	Time limit for online direct reporting after medical institutions identify suspected CHIK cases	653 (59.80)
Q15	Typical manifestations of CHIK in the chronic phase	1,000 (91.58)

**Table 4:** Cognition accuracy across domains of the three-level prevention framework (*N* = 1,092).

Three-level prevention dimensions	Average correct rate (%)
Primary prevention: etiological interruption	
Transmission routes (Q1) + sources of infection (Q8) + high-risk areas (Q9) + incubation period (Q11) + high-risk groups (Q13)	88.68
Secondary prevention: early identification	
Clinical manifestations (Q2) + conditions for suspected cases (Q3) + differential diagnosis (Q4) + diagnostic basis for confirmed cases (Q5)	67.74
Tertiary prevention: clinical management	
Isolation time limit (Q6) + treatment principles (Q7) + emergency measures (Q10) + chronic phase manifestations (Q15)	87.37
Monitoring support system	
Nucleic acid detection criteria (Q12) + reporting time limit (Q14)	51.19

**Table 5:** Total CHIK knowledge scores by respondent characteristics (*N* = 1,092).

Demographic characteristics	Total number	Knowledge score mean ± std.	95% CI for mean	Knowledge score median (Q1, Q3)	Mean rank	*U*/*H*	*p*
Gender						74016.500	0.243
Female	922	11.72 ± 2.05	(11.59, 11.86)	12.00 (11.00, 13.00)	551.22		
Male	170	11.32 ± 2.60	(10.93, 11.72)	12.00 (10.00, 13.00)	520.89		
Age						2.054	0.358
≤30 years	341	11.67 ± 2.25	(11.44,11.91)	12.00 (11.00, 13.00)	556.21		
31–40 years	513	11.58 ± 2.13	(11.40, 11.77)	12.00 (10.00, 13.00)	532.39		
≥41 years	238	11.82 ± 2.02	(11.56, 12.08)	12.00 (11.00, 13.00)	563.00		
Educational background						21.630	<0.001^a^
Junior college or below	157	11.10 ± 2.52	(10.70, 11.49)	12.00 (10.00, 13.00)	478.55		
Bachelor’s degree	788	11.64 ± 2.12	(11.50, 11.79)	12.00 (11.00, 13.00)	542.11		
Master’s degree or above	147	12.36 ± 1.57	(12.10, 12.62)	13.00 (11.00, 14.00)	642.59		
Job type						33.184	<0.001^b^
Doctor	415	12.11 ± 1.82	(11.94, 12.29)	12.00 (11.00, 13.00)	607.72		
Nurse	577	11.34 ± 2.19	(11.16, 11.52)	12.00 (10.00, 13.00)	495.50		
Others	100	11.64 ± 2.76	(11.09, 12.19)	12.00 (10.00, 14.00)	586.67		
Hospital level						60488.500	<0.001
Tertiary hospital	932	11.79 ± 2.04	(11.66, 11.92)	12.00 (11.00, 13.00)	561.60		
Secondary hospital	160	10.93 ± 2.57	(10.53, 11.33)	11.00 (10.00, 13.00)	458.55		
Hospital type						1.080	0.583
General hospital	567	11.59 ± 2.29	(11.40, 11.78)	12.00 (10.00, 13.00)	542.12		
Maternal and child specialist hospital	445	11.70 ± 1.98	(11.53, 11.90)	12.00 (10.00, 13.00)	545.92		
Other specialist hospitals	80	11.89 ± 1.99	(11.45, 12.33)	12.00 (11.00, 13.00)	580.72		
Department						26.173	<0.001^c^
High-risk clinical departments	421	11.85 ± 1.95	(11.67, 12.04)	12.00 (11.00, 13.00)	567.36		
Special population departments	319	11.99 ± 1.87	(11.78, 12.19)	12.00 (11.00, 13.00)	587.10		
General clinical departments	159	10.90 ± 2.45	(10.52, 11.28)	11.00 (10.00, 12.00)	443.77		
Non-clinical departments	193	11.34 ± 2.50	(10.98, 11.69)	12.00 (10.00, 13.00)	518.53		
Practice category						19.084	<0.001^d^
Western medicine	823	11.85 ± 1.98	(11.72, 11.99)	12.00 (11.00, 13.00)	569.19		
Public health	210	10.97 ± 2.59	(10.61, 11.32)	11.00 (9.00, 13.00)	465.40		
Others	59	11.49 ± 2.14	(10.93, 12.05)	12.00 (11.00, 13.00)	518.74		
Years of working experience						1.672	0.433
≤10 years	585	11.68 ± 2.20	(11.50, 11.85)	12.00 (11.00, 13.00)	553.86		
11–20 years	358	11.59 ± 2.08	(11.37, 11.81)	12.00 (10.00, 13.00)	529.23		
≥21 years	149	11.79 ± 2.09	(11.45, 12.12)	12.00 (10.00, 13.00)	559.10		
Professional title						5.176	0.159
Uncertified	42	11.57 ± 2.40	(10.82, 12.32)	12.00 (10.00, 14.00)	548.12		
Primary	398	11.48 ± 2.35	(11.25, 11.71)	12.00 (10.00, 13.00)	529.35		
Intermediate	496	11.70 ± 2.00	(11.53, 11.88)	12.00 (11.00, 13.00)	544.55		
Senior	156	12.03 ± 1.93	(11.72, 12.33)	12.00 (11.00, 14.00)	596.03		

a Post hoc tests (Bonferroni-corrected *α* = 0.0167) revealed that the Master’s or above group differed significantly from both the Associate or below (*U* = 8103.500, *p* < 0.001) and Bachelor’s (*U* = 47229.500, *p* < 0.001) groups. No significant difference was found between the Associate or below and Bachelor’s groups (*p* = 0.019).

b Post hoc comparisons using the Bonferroni correction (*α* = 0.0167) showed significant differences between physicians and nurses (*U* = 94666.000, *p* < 0.001) and between nurses and other staff (*U* = 24486.500, *p* = 0.015), but not between physicians and other staff (*U* = 20403.500, *p* = 0.792).

c Post hoc comparisons using the Bonferroni correction (*α* = 0.0083) identified two statistically significant differences: between high-risk and general clinical departments (*U* = 25740.500, *p* < 0.001) and between special populations and general clinical departments (*U* = 18664.500, *p* < 0.001). All other pairwise comparisons (high-risk vs. special populations, high-risk vs. non-clinical, special populations vs. non-clinical, and general clinical vs. non-clinical) were not statistically significant (*p* > 0.0083).

d Post hoc comparisons using the Bonferroni correction (*α* = 0.0167) identified a statistically significant difference only between western medicine physicians and public health physicians (*U* = 70061.500, *p* < 0.001). No significant differences were found between Western Medicine Physicians and Others (*U* = 21962.000, *p* = 0.214) or between Public Health Physicians and Others (*U* = 5516.500, *p* = 0.195).

### 3.4 Examination of attitudinal tendencies

**Figure 1:**
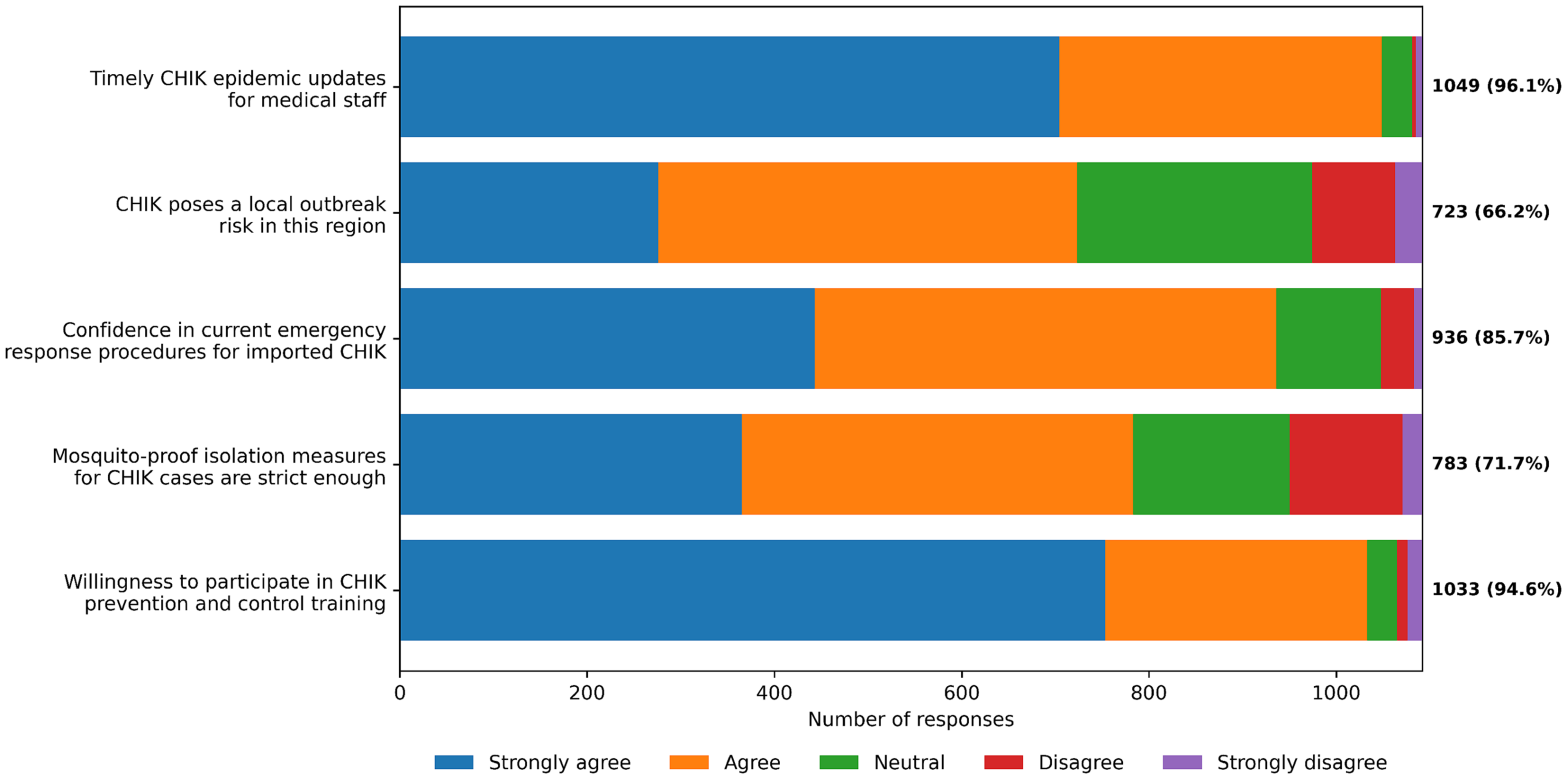
Distribution of perspectives on CHIK prevention and control among medical staff (*N* = 1,092). CHIK, chikungunya fever. Stacked bars show the number of responses across a 5-point Likert scale (strongly agree to strongly disagree). Numbers at the right indicate the combined total of “strongly agree” and “agree” for each statement.

The general sentiments were positive, with the majority of respondents expressing agreement with comments regarding CHIK prevention and control (Figure 1). The score range of respondents’ attitudes toward CHIK prevention and control was 5–25 points, with a mean of 21.08 ± 2.82, a median of 21.00 (19.00, 23.00). CHIK prevention-and-control attitude scores varied significantly by age, job role, hospital category, practice category, years in practice, and professional title. Respondents aged ≥41 years scored lower than those aged ≤30 years (*p* < 0.001). Scores from maternal and child health hospitals and children’s hospitals were lower than those from general hospital (*p* = 0.002) as well as other specialty hospitals (*p* = 0.007). Public health practitioners scored higher than western medicine practitioners (*p* < 0.001) other practice categories (*p* = 0.009). Those with ≥21 years in practice scored lower than those with 11–20 years (*p* = 0.016) and ≤10 years (*p* < 0.001). Respondents with senior title scored lower than those without a professional title (*p* < 0.001), those with primary title (*p* < 0.001) and with intermediate title (*p* = 0.003) (Table 6).

**Table 6:** Attitude scores toward CHIK prevention and control by respondent characteristics (*N* = 1,092).

Demographic characteristics	Total number	Attitude score mean ± std.	95% CI for mean	Attitude score median (Q1, Q3)	Mean rank	*U*/*H*	*p*
Gender						77109.000	0.737
Female	922	21.10 ± 2.70	(20.93, 21.28)	21.00 (19.00, 23.00)	545.13		
Male	170	20.98 ± 3.37	(20.47, 21.49)	21.00 (19.00, 24.00)	553.92		
Age						15.176	<0.001^a^
≤30 years	341	21.40 ± 3.03	(21.08, 21.72)	22.00 (20.00, 24.00)	591.67		
31–40 years	513	21.08 ± 2.69	(20.85, 21.31)	21.00 (19.00, 23.00)	543.16		
≥41 years	238	20.63 ± 2.71	(20.28, 20.97)	21.00 (19.00, 23.00)	488.98		
Educational background						3.500	0.174
Junior college or below	157	21.38 ± 3.10	(20.89, 21.86)	21.00 (20.00, 25.00)	586.12		
Bachelor’s degree	788	21.05 ± 2.82	(20.85, 21.25)	21.00 (19.00, 23.00)	543.18		
Master’s degree or above	147	20.95 ± 2.46	(20.54, 21.35)	21.00 (19.00, 23.00)	522.00		
Job type						6.914	0.032^b^
Doctor	415	20.92 ± 2.74	(20.66, 21.18)	21.00 (19.00, 23.00)	528.58		
Nurse	577	21.28 ± 2.83	(21.05, 21.51)	21.00 (19.00, 24.00)	568.38		
Others	100	20.59 ± 3.00	(20.00, 21.18)	20.00 (19.00, 23.00)	494.65		
Hospital level						70062.000	0.219
Tertiary hospital	932	21.06 ± 2.74	(20.88, 21.23)	21.00 (19.00, 23.00)	541.67		
Secondary hospital	160	21.23 ± 3.22	(20.73, 21.73)	21.00 (19.00, 24.00)	574.61		
Hospital type						13.137	0.001^c^
General hospital	567	21.25 ± 2.88	(21.02, 21.49)	21.00 (19.00, 24.00)	569.17		
Maternal and child specialist hospital	445	20.77 ± 2.72	(20.52, 21.02)	21.00 (19.00, 23.00)	506.73		
Other specialist hospitals	80	21.60 ± 2.79	(20.98, 22.22)	22.00 (20.00, 24.00)	607.07		
Department						1.767	0.622
High-risk clinical departments	421	21.11 ± 2.75	(20.85, 21.38)	21.00 (19.00, 23.00)	547.30		
Special population departments	319	21.13 ± 2.71	(20.84, 21.43)	21.00 (19.00, 23.00)	550.80		
General clinical departments	159	21.21 ± 2.94	(20.75, 21.67)	21.00 (19.00, 24.00)	565.05		
Non-clinical departments	193	20.82 ± 3.02	(20.40, 21.25)	21.00 (19.00, 23.00)	522.37		
Practice category						13.568	0.001^d^
Western medicine	823	20.99 ± 2.69	(20.81, 21.18)	21.00 (19.00, 23.00)	532.48		
Public health	210	21.61 ± 3.07	(21.19, 22.03)	22.00 (20.00, 24.00)	615.93		
Others	59	20.42 ± 3.30	(19.56, 21.28)	21.00 (19.00, 23.00)	494.90		
Years of working experience						18.852	<0.001^e^
≤10 years	585	21.34 ± 2.89	(21.10, 21.57)	21.00 (20.00, 24.00)	578.96		
11–20 years	358	20.97 ± 2.63	(20.69, 21.24)	21.00 (19.00, 23.00)	529.75		
≥21 years	149	20.36 ± 2.80	(19.90, 20.81)	20.00 (18.00, 22.00)	459.31		
Professional title						27.936	<0.001^f^
Uncertified	42	22.02 ± 2.93	(21.11, 22.94)	22.00 (20.00, 25.00)	659.51		
Primary	398	21.38 ± 2.97	(21.09, 21.67)	21.00 (20.00, 24.00)	587.40		
Intermediate	496	21.01 ± 2.71	(20.77, 21.25)	21.00 (19.00, 23.00)	534.63		
Senior	156	20.29 ± 2.51	(19.90, 20.69)	20.00 (18.00, 22.00)	449.47		

a Post hoc pairwise comparisons with a Bonferroni correction (*α* = 0.0167) identified a statistically significant difference only between the ≤30 and ≥41 age groups (*U* = 33042.000, *p* < 0.001). The comparisons between the ≤30 and 31–40 groups (*U* = 79601.000, *p* = 0.025) and between the 31–40 and ≥41 groups (*U* = 54894.000, *p* = 0.025) were not statistically significant.

b Post hoc pairwise comparisons with Bonferroni correction (*α* = 0.0167) showed no statistically significant differences between any groups (all *p* > 0.0167).

c Post hoc tests comparisons with Bonferroni-corrected (*α* = 0.0167) revealed significant differences between General and Maternal & Child Hospitals (*U* = 111790.500, *p* = 0.002) and between Maternal & Child and Other Specialty Hospitals (*U* = 14467.500, *p* = 0.007). All other comparisons were not significant.

d Post hoc pairwise comparisons with Bonferroni correction (*α* = 0.0167) revealed significant differences between Western Medicine and Public Health Physicians (*U* = 73194.500, *p* < 0.001) and between Public Health Physicians and Others (*U* = 4835.000, *p* = 0.009). All other comparisons were not significant.

e Post hoc tests (Bonferroni-corrected *α* = 0.0167) revealed that the ≥21 years group differed significantly from both the ≤10 years (*U* = 34192.500, *p* < 0.001) and 11–20 years groups (*U* = 23069.000, *p* = 0.016). No significant difference was found between the ≤10 and 11–20 years groups (*p* = 0.017).

f Post hoc comparisons (Bonferroni-corrected *α* = 0.0083): No Title vs. Senior: *U* = 2044.000, *p* < 0.001—significant. Junior vs. Senior: *U* = 23240.500, *p* < 0.001—significant. Intermediate vs. Senior: *U* = 32587.500, *p* = 0.003—significant. All other pairwise comparisons: not significant (*p* > 0.0083).

### 3.5 Evaluation of training efficacy

An examination of the overall knowledge scores of medical personnel who underwent CHIK training compared to those who did not indicated that the taught cohort exhibited superior knowledge scores relative to the untrained cohort (Table 7), suggesting better cognition among trained participants.

**Table 7:** Total knowledge scores by CHIK training exposure (*N* = 1,092).

CHIK training participation	Knowledge score mean ± std.	Knowledge score median (Q1, Q3)	Mean rank	*U*	*p*
Trained group (*N* = 914)	11.76 ± 2.11	12.00 (11.00, 13.00)	579.88	50837.500	<0.001
Untrained group (*N* = 178)	11.14 ± 2.27	11.00 (10.00, 13.00)	375.10		

### 3.6 Determinants of medical personnel’s attitudes toward CHIK prevention and control

A hierarchical (block-wise) logistic regression analysis was employed to investigate the determinants of medical personnel’s attitudes toward CHIK prevention and control. The dependent variable was the positivity of the prevention and control attitude (defined in the Materials and Methods), whereas the independent factors were incorporated into the model in three phases: (1) Block 1: Demographic attributes (gender, age, educational qualifications, occupation, hospital tier, hospital classification, department, practice category, professional designation); (2) Block 2: Involvement in CHIK training; (3) Block 3: CHIK-related knowledge assessment score. The model fitting findings indicated *χ*^2^ = 106.493 (*p* < 0.001), demonstrating the final model’s significance, with a Nagelkerke *R*^2^ = 0.135 (Table 8).

**Table 8:** Hierarchical logistic regression model fit across blocks (outcome: positive attitude).

Block	Added variables	Model significance (omnibus test)		Model fit (Nagelkerke *R*^2^)
		*χ^2^*	*p*	
1	Demographic characteristics	30.357	0.024	0.040
2	Training status	94.103	<0.001	0.120
3	Knowledge score	106.493	<0.001	0.135

In summary, training exposure and elevated knowledge scores were the most significant independent correlates of a favorable prevention-and-control attitude. Respondents who had received CHIK training had higher odds than those without training (OR = 3.971, *p* < 0.001). Each 1-point increase in total CHIK knowledge score was associated with a 13% increase in the odds of a positive attitude (OR per point = 1.130, *p* < 0.001) (Table 9).

**Table 9:** Factors associated with positive attitudes toward CHIK prevention and control (hierarchical logistic regression).

Variable	*β*	S.E.	Wald	*p*	OR (95% CI)
Gender (male)	0.116	0.214	0.294	0.587	1.123 (0.738~1.708)
Age (years)	−0.028	0.015	3.462	0.063	0.973 (0.945~1.001)
Educational background			3.197	0.202	
Junior college or below			Ref		
Bachelor’s degree	−0.056	0.232	0.057	0.811	0.946 (0.600~1.492)
Master’s degree or above	0.389	0.340	1.310	0.252	1.475 (0.758~2.872)
Job type			6.201	**0.045**	
Doctor			Ref		
Nurse	0.173	0.232	0.723	0.395	1.189 (0.798~1.770)
Others	−0.584	0.316	3.418	0.064	0.558 (0.300~1.036)
Hospital level (secondary)	0.040	0.239	0.028	0.867	1.041 (0.651~1.663)
Hospital type			1.547	0.461	
General hospital			Ref		
Maternal and child specialist hospital	−0.126	0.165	0.584	0.445	0.881 (0.638~1.218)
Other specialist hospitals	0.245	0.323	0.574	0.448	1.277 (0.679~2.403)
Department			0.938	0.816	
High-risk clinical departments			Ref		
Special population departments	0.144	0.188	0.585	0.444	1.155 (0.798~1.671)
General clinical departments	0.193	0.245	0.621	0.431	1.213 (0.751~1.958)
Non-clinical departments	0.145	0.241	0.360	0.548	1.156 (0.720~1.854)
Practice category			3.753	0.153	
Western medicine			Ref		
Public health	0.415	0.217	3.648	0.056	1.514 (0.989~2.317)
Others	−0.025	0.326	0.006	0.939	0.975 (0.515~1.847)
Professional title			3.239	0.356	
Uncertified			Ref		
Primary	−0.599	0.465	1.661	0.198	0.549 (0.221~1.366)
Intermediate	−0.819	0.487	2.824	0.093	0.441 (0.170~1.146)
Senior	−0.818	0.570	2.061	0.151	0.441 (0.144~1.348)
CHIK training received	1.379	0.182	57.476	**<0.001**	3.971 (2.780~5.673)
Total CHIK knowledge score	0.122	0.035	12.394	**<0.001**	1.130 (1.056~1.210)

Bold values indicate statistically significant results (*p* < 0.05).

### 3.7 Relationship between attitude and knowledge

The correlation between knowledge and attitude was positive but weak (*r* = 0.119, *p* < 0.001; 95% CI 0.060–0.177). The cumulative knowledge score represented about 1.4% of the variance in the overall attitude score (*R*^2^ = 0.014), signifying a minimal effect size. Curve estimation revealed no indication of a non-linear relationship (quadratic term *p* = 0.257; cubic term *p* = 0.495), and there was no significant enhancement in model fit (*R*^2^), indicating a poor linear correlation between knowledge and attitudes.

## 4 Discussion

This research encompassed 20 medical institutes in Chengdu and involved 1,092 healthcare professionals. The sample predominantly consisted of nurses and doctors, featuring a significant female majority and a notable presence from tertiary hospitals, specifically in emergency departments, fever clinics, and maternity and child health departments. It may reasonably represent the present condition of early detection and treatment of imported mosquito-borne infections in major metropolitan general and specialist hospitals. This sample structure aligns with the local context of CHIK prevention and management, offering a dependable foundation for further research on knowledge, attitudes, and their determinants. In contrast to previous reports that mainly focus on imported cases or outbreaks, our study enhances public health knowledge by assessing the preparedness of frontline medical personnel in a non-endemic inland metropolitan area and identifying specific operational cognitive deficiencies directly associated with early detection and surveillance measures (e.g., RT-PCR Ct interpretation and the required reporting timeline). This addresses an evidence deficiency for Chengdu/Sichuan, where systematic evaluations were previously absent, and offers actionable objectives for hospital preparedness planning and risk communication.

Training is a variable system-level aspect and hence pivotal to preparation; nonetheless, its interpretation must be grounded in the methodology employed in this survey. The study indicated that the overall training coverage for CHIK among medical personnel was high, attaining 83.70%, likely correlated with the enhanced regional early warning systems and reinforced in-hospital training subsequent to the imported CHIK incident in Foshan, China, in July 2025. Moreover, our findings revealed a disparity in training participation among various categories. The training participation rate among staff with a master’s degree or higher was inferior to that of bachelor’s degree holders, potentially attributable to their self-assessment of “having already mastered the knowledge,” restricted time commitment, or institutional presuppositions that this demographic already possesses foundational knowledge. Nonetheless, they exhibited shortcomings in areas such as laboratory result interpretation, indicating that academic qualifications alone cannot substitute for practical clinical training. Yang et al. (16) emphasized the necessity of enhancing specialized technical training for clinical personnel to augment their diagnostic and emergency response skills for imported cases, suggesting that highly educated groups also require institutionalized training to address deficiencies in the intricacies of diagnosis and treatment procedures. The training participation rate of secondary hospitals was inferior to that of tertiary hospitals; nonetheless, secondary hospitals frequently serve as the initial consultation point for imported cases. The insufficient resources and teaching personnel may result in the delayed detection and reporting of CHIK in its initial phase. A study by Tian et al. (17) indicated that the awareness and reporting acumen of attending physicians must be enhanced; inadequate training in primary and secondary institutions may result in overlooked identification and delayed reporting, hence elevating the danger of local transmission. The training involvement rate was lowest among junior medical personnel without professional titles, as their daily responsibilities primarily involve fundamental clinical duties. The training on infectious disease protocols is condensed, potentially resulting in diminished personal preventive and control competencies over time. Consequently, training should be conducted hierarchically according to educational attainment, hospital classification, and professional designation, emphasizing the enhancement of critical components such as diagnostic criteria, online direct reporting, and mosquito-proof isolation. The training in this investigation was assessed as a binary self-reported exposure (“ever received CHIK-related training”) lacking precise information regarding the nature, date, duration, or location of the instruction. Consequently, variations in training participation among subgroups should be regarded as descriptive trends rather than indicators of the efficacy of particular training modalities. This measurement constraint suggests that “training” may partially indicate broader institutional preparedness (e.g., availability of educational resources, administrative focus) rather than a standardized intervention, and we refrain from over-interpreting it as a completely defined risk factor.

Prior research has indicated that the medical personnel’s awareness of CHIK is suboptimal. A paper by Simon et al. (9) indicated that insufficient awareness may result in missed diagnoses and underreporting; findings by Gomes de Azevedo-Quintanilha et al. (18) and Montalvo Zurbia-Flores et al. (19) similarly demonstrated that in non-endemic regions, symptoms including severe joint pain and fever are frequently misdiagnosed as other illnesses. From a risk management standpoint, the most significant gaps are those that can postpone recognition, confirmation, and obligatory reporting, thereby extending the period for secondary transmission. This study consistently demonstrated that, while the overall knowledge level was above average, significant deficiencies were evident in critical areas: only 42.58% accurately understood the nucleic acid detection threshold, 54.85% could define suspected cases, 56.14% comprehended the diagnostic criteria for confirmed cases, and 59.80% recognized the necessity for 24 h online direct reporting of suspected cases. These elements signify high-risk operational stages in the surveillance process, and inaccuracies at these junctures may result in postponed reporting and deferred vector-control measures in a non-endemic environment. These vulnerabilities directly align with the decision-making roles within the management hierarchy, suggesting that “understanding the fundamentals” has not yet consistently manifested as “adhering to established standards.” This aligns with Zhen et al.’s (10) focus on “joint prevention and control, early diagnosis, and standardized management,” indicating that training should emphasize early detection and procedural implementation. Additionally, Tian et al.’s (17) caution regarding the similar early symptoms of mosquito-borne diseases, which can lead to misdiagnosis, reflects the ambiguity surrounding case definitions and reporting timelines in this study, underscoring the necessity to enhance differential diagnosis of various pathogens and critical process points. Despite the explicit case definitions and reporting requirements outlined in the Guidelines for the Prevention and Control of Chikungunya Fever (2025 Edition) (20), a disconnect persists between “cognition and details” in frontline execution, particularly regarding laboratory result interpretation and direct reporting connections. It is imperative to transform conventional phrases into job-specific and contextualized checklists and evaluations. Stratified analyses indicated a distinct structural gradient in knowledge based on education, employment function, hospital level, and department, aligning with variations in exposure and access to continuing education opportunities. This pattern aligns with the frequency of exposure, availability of continuing education options, and the significance of clinical expertise in the questionnaire. In conjunction with the research conducted by Chen et al. (11) regarding the seasonal and regional clustering of cases (predominantly observed in late summer and autumn, with localized transmission more probable in Yunnan, Guangdong, and Zhejiang), it can be deduced that advanced hospitals and high-exposure departments possess greater access to educational and practical opportunities. Conversely, secondary institutions and general departments ought to deliver focused supplementary training on “error-prone points,” including nucleic acid threshold interpretation, differentiation between suspected and confirmed criteria, and 24 h direct reporting, to mitigate the knowledge disparity arising from variations in experience and resources.

This study demonstrated that medical personnel predominantly exhibited favorable attitudes toward CHIK prevention and control, indicating a strong acknowledgment of the importance of prevention and control and a readiness to engage. Consequently, we concentrated on determining the modifiable and non-modifiable characteristics linked to a favorable prevention-and-control mindset, to guide specific preparedness initiatives. Consistent patterns emerged across various groups: younger employees and those with limited work experience exhibited greater positivity, likely attributable to their expedited access to information, heightened risk awareness, and increased receptivity to institutional changes; nurses outperformed doctors, potentially due to their involvement in frontline operations and direct exposure to the practical implications of policies; public health professionals scored higher than their Western medicine counterparts, aligning with their prevention-focused professional ethos; staff lacking professional titles achieved higher scores than those with intermediate or senior titles, indicating that lower-title groups are more attentive to norms and procedures. This pattern aligns with the findings of Harun et al. (21): nurses exhibited greater willingness to participate in prevention and control efforts compared to doctors, public health personnel surpassed clinical doctors, and younger, less experienced staff were more inclined than their senior counterparts, thereby offering external validation for the interpretation of this study. In accordance with this approach, multivariable analysis was employed to elucidate the independent relationships between training and knowledge and demographic/occupational factors. The findings from stratified logistic regression indicated that the primary determinants influencing medical personnel’s attitudes toward CHIK prevention and control were derived from job characteristics, training experience, and knowledge base: job type, training participation, and knowledge score were all included in the final model; the likelihood of possessing a positive attitude among trained personnel was 3.971 times greater than that of untrained personnel, and for each 1-point increment in knowledge score, the probability of a positive attitude increased by 13.0%. Incorporating the training variable enhanced the model’s explanatory power from 0.040 to 0.120, signifying that training had the most substantial impact on improving attitudes; this conclusion aligns with the findings of El Sebaey et al. (22) and Zhang et al. (23), which suggest that training facilitates the conversion of knowledge into a proactive prevention attitude. Simultaneously, knowledge and attitude exhibited a weak positive correlation at the bivariate level, with knowledge demonstrating limited explanatory power for variations in attitude. The data suggests that attitude is influenced by various factors, including organizational support, process operability, and individual practical experience (22, 23). Consequently, training is efficacious for two reasons: first, it rectifies the under-appreciation of risks in non-endemic regions via risk communication; second, it elucidates job responsibilities and enhances self-efficacy through standardized process training, thus progressing from “knowing what should be done” to “being willing and able to execute it.”

This study possesses certain drawbacks. In the updated Discussion, we concentrate on findings that directly correspond to the study objectives (cognition gaps pertinent to readiness and factors influencing attitudes) and refrain from presenting further descriptive results that are appropriate for the Results section. The sample’s representativeness is constrained: the survey predominantly originated from 20 institutions, primarily tertiary hospitals and clinical roles, and did not adequately encompass community health service centers and township health centers. A selection bias exists, favoring those more inclined to participate; hence, caution is warranted when generalizing the findings to the entire primary physician staff. Secondly, the cross-sectional design fails to establish a causal relationship; the correlation between training and attitude may exhibit reverse causality. Furthermore, this study did not directly observe behavioral indicators, such as actual mosquito-proof isolation operations, specimen submission, and reporting behaviors, thereby complicating the comprehensive representation of the transmission chain among knowledge, attitude, and behavior. Future research may monitor alterations in knowledge, attitudes, and behaviors pre- and post-training via prospective cohorts or phased intervention assessments, broaden the sample to include primary institutions, and incorporate objective process indicators and significant confounding variables to yield evidence with enhanced explanatory strength and generalizability.

Medical workers in Chengdu exhibited generally proficient knowledge regarding CHIK and maintained positive attitudes toward prevention and control; nevertheless, significant deficiencies persist in early detection, RT-PCR nucleic acid interpretation, and prompt online reporting. Training correlated with enhanced attitudes; yet, mere knowledge enhancement may not guarantee enduring behavioral modification. It is advisable to implement standardized, process-oriented, and hierarchical training, bolstered by unified protocols and data-driven reporting systems, to enhance preparation in non-endemic hospitals confronting persistent importation concerns.

## Data Availability

The raw data supporting the conclusions of this article will be made available by the authors, without undue reservation.
